# Revisiting the Palimpsest: Exploring the Interaction of the Variance and Structure of Developmental Determinants of Phenotypic Covariance

**DOI:** 10.1093/icb/icag111

**Published:** 2026-07-07

**Authors:** Fernando Andrade, J David Aponte, Nathan M Young, Ralph S Marcucio, Benedikt Hallgrímsson

**Affiliations:** Alberta Children’s Hospital Research Institute, University of Calgary, Calgary, Alberta T2N 4N1, Canada; McCaig Institute for Bone and Joint Health, Calgary, University of Calgary, Calgary, Alberta T2N 4N1, Canada; Libin Cardiovascular Institute, University of Calgary, Calgary, Alberta T2N 4N1, Canada; Libin Cardiovascular Institute, University of Calgary, Calgary, Alberta T2N 4N1, Canada; Department of Orthopedic Surgery, University of California San Francisco, San Francisco, California 94110, United States; Alberta Children’s Hospital Research Institute, University of Calgary, Calgary, Alberta T2N 4N1, Canada; Department of Cell Biology & Anatomy, University of Calgary, Calgary, Alberta T2N 4N1, Canada

## Abstract

The analysis of patterns of trait covariance is of tremendous importance in the study of evolution and development as well as in quantitative genetics. Multivariate quantitative genetics and morphological integration were combined in the 1980s under the latent variable model for integration developed by Wagner and Cheverud. The palimpsest framework builds on this model. Based on a definition of integration as disposition, it distinguishes between the developmental determinants of covariation (integration) and observed phenotypic covariance patterns. Further, the palimpsest distinguishes between the role of the distribution of variance across latent processes that generate covariance versus the developmental architectural features that connect those processes to phenotypic traits. In this paper, we construct a simple simulation to illustrate how these two different aspects of integration relate to observed phenotypic covariance patterns. We show that: (1) changes in the distribution of variance across latent processes can significantly alter both integratedness and covariance structure, (2) increases in the variance of leading processes tend to increase integratedness and the circumstances under which integratedness decreases are more restricted, (3) changes in the relative variances of latent processes can result in discontinuous change covariance structure, (4) changes in which developmental processes are linked to which traits (the process–trait map) can also alter covariance structure and interact with how variance is allocated among processes in complex ways. These findings can serve to inform and ground hypotheses about covariance structures and their interpretation in real biological settings.

## Introduction

“Hairless dogs have imperfect teeth; long-haired and coarse-haired animals are apt to have, as is asserted, long or many horns; pigeons with feathered feet have skin between their outer toes; pigeons with short beaks have small feet, and those with long beaks large feet. Hence, if man goes on selecting, and thus augmenting, any peculiarity, he will almost certainly unconsciously modify other parts of the structure, owing to the mysterious laws of the correlation of growth.” (Darwin 1859, p. 18)

 

“From the conception that life is the harmonious expression of a collection of phænomena regularly conditioned, it follows that the activity of an organ cannot be regarded as really existing for itself alone. Every kind of arrangement presupposes a series of other arrangement; every organ, therefore, must have intimate relations with the rest, and be more or less dependent on others.” (Gegenbaur 1878, p. 58)

 

“It is easy to see that co-relation must be the consequence of the variations of the two organs being partly due to common causes.” (Galton 1889, p. 135)

Morphological integration is a core concept linking considerations of evolution and development. This concept, which captures the tendency for traits to covary, connects to ideas about bias and constraint in the generation of variation as well as the effective dimensionality of phenotypic variation (Simpson 1953; Hallgrímsson et al. 2023. In earlier work, we proposed the Palimpsest model to study the developmental determinants of phenotypic covariance structures (Hallgrimsson et al. 2009). The model treats organismal development as a series of interacting steps in which each builds on, modifies, and acts as a substrate for what follows. In craniofacial development, for example, neural crest-related variation affects primarily facial and anterior cranial traits, whereas later brain growth contributes variation to the vault and additional landmarks. These processes act at different times and map onto different trait subsets, yet jointly shape covariance structure. Unfortunately, this model is much misunderstood, presented variously as a problem (“covariance is complicated”) or as a specific model to test (“covariance structure has multiple overlapping determinants”). In fact, the Palimpsest model is neither of these things. Instead, it is a conceptual framework for the analysis of covariance structure that is premised on two fundamental distinctions. The first is the separation of observed covariance from the developmental determinants of covariance. The Palimpsest model does this by redefining integration as a dispositional concept, following Wagner’s redefinition of canalization (Wagner et al. 1997). This definition allows for the possibility that similar underlying determinants, states of integration, can create different patterns of covariance under different conditions. This leads to the second distinction which is between the role of variation in developmental determinants and the relationship of those determinants to each other. The palimpsest model predicts that covariance structures can be influenced by both the distribution of variance across the developmental determinants of covariation and the ways in which those determinants map on to phenotypic traits. It thus distinguishes between the flow of variation through developmental processes and the topology of the mapping of those processes on to traits. The initial papers test this idea by showing how changes in the variance of specific processes or, more generally via inbreeding, can dramatically alter covariance structure (Hallgrimsson et al. 2006; Hallgrimsson et al. 2009; Jamniczky and Hallgrimsson 2009). This is important because differences in covariance structure are often interpreted in terms of inference about modularity or the topology of process to trait mappings—for example, when shifts in cranial correlation structure across mammals are read as shifts in modular organization among traits (Goswami 2006). Within microevolutionary contexts, however, such topologies tend to be relatively invariant whereas the distribution of variance across covariance generating processes can be relatively easily changed.

Morphological integration is a core concept linking considerations of evolution and development. This concept, which captures the tendency for traits to covary, connects to ideas about bias and constraint in the generation of variation as well as the effective dimensionality of phenotypic variation (Simpson 1953; Hallgrímsson et al. 2023). In earlier work, we proposed the Palimpsest model to study the developmental determinants of phenotypic covariance structures (Hallgrimsson et al. 2009). The model treats organismal development as a series of interacting steps in which each builds on, modifies, and acts as a substrate for what follows. In craniofacial development, for example, neural crest-related variation affects primarily facial and anterior cranial traits, whereas later brain growth contributes variation to the vault and additional landmarks. These processes act at different times and map onto different trait subsets, yet jointly shape covariance structure. Unfortunately, this model is much misunderstood, presented variously as a problem (“covariance is complicated”) or as a specific model to test (“covariance structure has multiple overlapping determinants”). In fact, the Palimpsest model is neither of these things. Instead, it is a conceptual framework for the analysis of covariance structure that is premised on two fundamental distinctions. The first is the separation of observed covariance from the developmental determinants of covariance. The Palimpsest model does this by redefining integration as a dispositional concept, following Wagner’s redefinition of canalization (Wagner et al. 1997). This definition allows for the possibility that similar underlying determinants, states of integration, can create different patterns of covariance under different conditions. This leads to the second distinction which is between the role of variation in developmental determinants and the relationship of those determinants to each other. The palimpsest model predicts that covariance structures can be influenced by both the distribution of variance across the developmental determinants of covariation and the ways in which those determinants map on to phenotypic traits. It thus distinguishes between the flow of variation through developmental processes and the topology of the mapping of those processes on to traits. The initial papers test this idea by showing how changes in the variance of specific processes or, more generally via inbreeding, can dramatically alter covariance structure (Hallgrimsson et al. 2006; Hallgrimsson et al. 2009; Jamniczky and Hallgrimsson 2009). This is important because differences in covariance structure are often interpreted in terms of inference about modularity or the topology of process to trait mappings—for example, when shifts in cranial correlation structure across mammals are read as shifts in modular organization among traits (Goswami 2006). Within microevolutionary contexts, however, such topologies tend to be relatively invariant whereas the distribution of variance across covariance generating processes can be relatively easily changed.

Virtually every phenotypic covariance structure in a real biological setting is a palimpsest in the sense that there are multiple developmental determinants for that structure and that those determinants have some kind of variation. It does not make sense, therefore, to test whether a particular covariance structure is a palimpsest but it is, hopefully, useful to apply the framework of the palimpsest to understand specific covariance structures and differences among them in evolutionary or experimental contexts. A critical gap in the initial presentation of the model is that we did not quantify how variance allocation among developmental processes and process–trait organization interact when both can shape the same covariance matrix. Here, we address this gap with controlled simulations built from a process–trait connection matrix D (topology) and a latent variance matrix Z. As a simplified illustration, mammalian bones can be thought of as arising through two broad forming processes, endochondral ossification and intramembranous ossification. Z captures how much endochondral ossification and intramembranous ossification each vary. D carries a different kind of information. It tells us which developmental process is responsible for variation in each trait. In a simplified D matrix, the entry for endochondral ossification would link that process to every trait it helps to form, long-bone lengths and cranial-base dimensions, while leaving other traits unconnected. The entry for intramembranous ossification would do the same for the membrane-formed skeleton, linking that process to calvarial and facial bones and to elements such as parts of the scapula, pelvis, and clavicle, but not to the bones assigned to endochondral growth.

With D and Z defined in this way, we generate phenotypic covariance matrices under alternative wirings and variance allocations, then ask how those matrices respond when variance in one developmental process is changed under a fixed protocol. In the analyses reported here, D is held constant within each scenario while the variance of a single latent process is scaled and the induced change in covariance is summarized in both the eigenvalue spectrum and the orientation of dominant trait-space directions. Consistent with the palimpsest framework, manifest covariance can change through two nonexclusive routes, by altering how much variance flows through a given process (changes in Z) or by altering how processes are connected to traits (changes in D), and these routes interact—the effect of scaling variance in one process depends on the underlying D map. In particular, regimes in which leading eigenvalues are weakly separated (often associated with more even allocation of variance across latent processes) exhibit greater instability of dominant directions, such that small variance changes can produce abrupt reordering and rotation of the leading subspace. We also show that the variance of eigenvalues (“integratedness”) is not a simple proxy for the heterogeneity of latent variances: it can emerge even under relatively uniform latent variance regimes once mapped through D, and decreases in eigenvalue dispersion are comparatively rare under one-at-a-time variance perturbations, making them especially informative when observed. Together, these perturbation responses—summarized by eigenvalue dispersion, effective dimensionality, and leading-subspace orientation—provide coarse constraints on which combinations of process variance and process–trait wiring remain compatible with observed patterns of covariance change. They also clarify how developmental architecture shapes the stability and evolvability of multivariate phenotypes.

## Methods

**Fig. 1:**
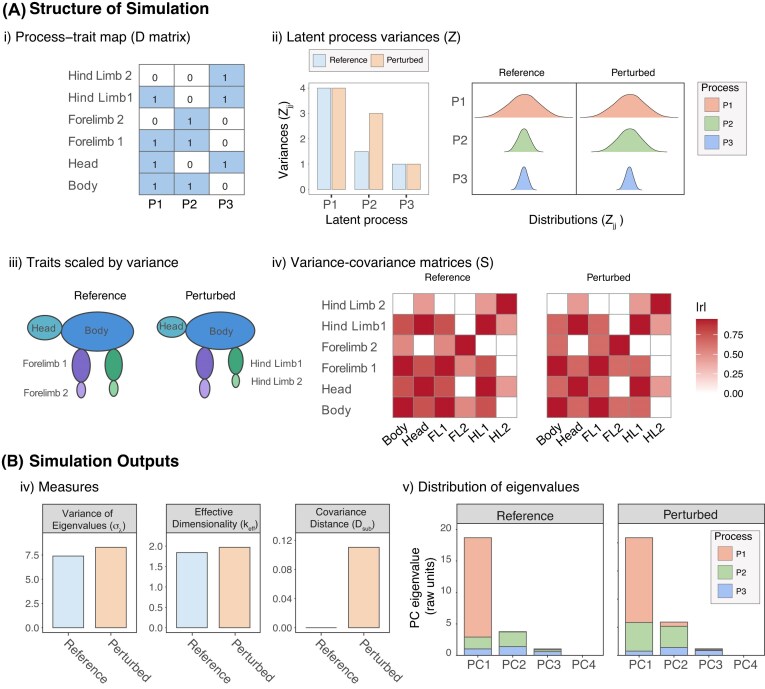
Workflow overview for the simulation and analysis. Panel A shows how each scenario is specified and how a perturbation is applied. (i) A process-trait map *D* links latent developmental processes (P1–P3) to traits (rows); entries indicate which processes contribute to which traits. (ii) A reference set of latent process variances *Z* is defined and a perturbation is introduced by changing one process variance while holding the rest fixed; the density sketches illustrate the implied change in each process distribution. (iii) Given *D* and *Z*, the perturbation changes the variance delivered to traits according to their loadings, altering the pattern of shared and trait-specific variation. (iv) These ingredients produce a reference and a perturbed trait variance-covariance matrix *S* (heatmaps). Panel B shows the summaries extracted from *S* that we use throughout the paper. (iv) We quantify changes between the reference and perturbed matrices using three complementary measures: *σ_λ_* (eigenvalue dispersion), *k_eff_* (effective spectral dimensionality), and *d*_sub_ (reorientation of the leading principal subspace). (v) We also visualize how the perturbation reshapes the eigenvalue spectrum of *S* (principal variances), linking changes in the three summaries to shifts in the distribution of variation across trait-space directions.

**Fig. 2:**
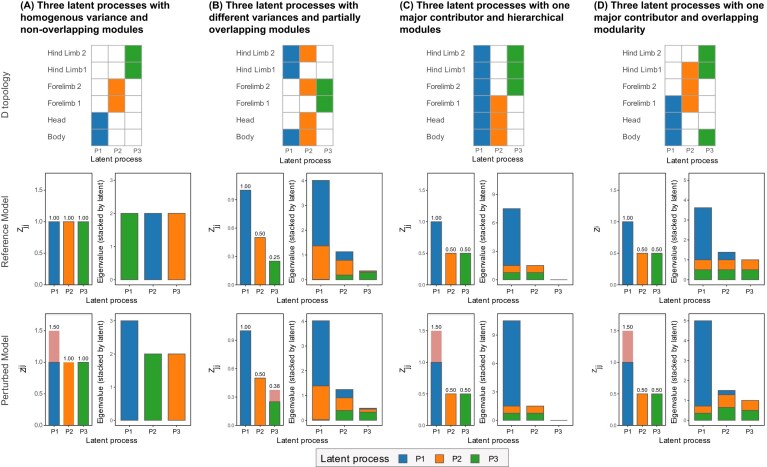
Example scenario types generated by combining a process-trait map (*D* topology) with a latent variance profile (*Z*) and applying a single-process variance perturbation. Columns A–D illustrate four representative configurations with three latent processes (P1–P3) and six traits. Top row: the process-trait map *D* (colored cells indicate which latent process contributes to each trait), highlighting differences in modularity, overlap, and hierarchical coupling. Middle row (reference model): the baseline latent process variances (left; *Z_jj_* for P1–P3) and the resulting contribution of each process to the eigenvalue spectrum of the trait covariance matrix (right; eigenvalues stacked by latent process). Bottom row (Perturbed model): the same quantities after perturbing one latent variance while holding the map fixed, showing how the change in *Z* propagates through *D* to reshape the spectrum. (A) Homogeneous latent variances with nonoverlapping modules. (B) Unequal latent variances with partially overlapping modules. (C) One major variance contributor combined with a hierarchical map. (D) One major variance contributor combined with an overlapping modular map.

The road map for the analysis in this work is summarized in Fig. 1. Fig. 2 illustrates four representative scenario types—combinations of process–trait map (*D*), latent variance allocation (*Z*), and a single-process perturbation—developed in the subsections below. We hold the process–trait layout fixed, vary the scale of variation on one developmental process at a time, and track how the induced trait covariation and the associated summary measures change between a reference and a perturbed state. The subsections that follow specify each step in turn.

### Trait covariance matrix—${\boldsymbol{S}}$

Here, we define how the trait covariance matrix $S\ $is built from the variance along each latent developmental process and from a binary map linking those processes to traits. We define $p\ $traits whose covariance arises from shared $k\ $latent developmental processes. The process-trait map is a $D\in\,{{\mathbb{R}}^{p \times k}}$ matrix map: each column specifies how each trait loads on the latent developmental processes (entries are binary). $Z\in\,{{\mathbb{R}}^{k \times k}}$ is the covariance matrix of the latent developmental variables. $Z\ $is a diagonal matrix, so ${{Z}_{jj}}$ is the variance contributed by process*j* before mapping to traits. Under a standard linear latent model, the covariance of traits is


\begin{eqnarray*}
S = DZD^{\top}.
\end{eqnarray*}

In this work, we fix two values of *k*, 3 and 4, while $p = 6\ $.

### ${\boldsymbol{D\ }}$ topologies

In this section we define the five binary process-trait maps that will be used across our analyses. Each column of $D\ $is a support vector indicating which traits are affected by that developmental process. We define five deterministic maps ${{D}_1},...,{{D}_5}$:

- Hierarchical (${{D}_1}$): nested supports (one process affects all traits; subsequent one get more restrictive).
- Modular overlapping (${{D}_2}$): three modules whose supports overlap so that no single process covers all traits.
- Modular nonoverlapping (${{D}_3}$): a partition of traits into three disjoint blocks (one process per block).
- Hybrid hierarchical + modular overlapping (${{D}_4}$): one column affects all traits; the other two columns define overlapping modular structure on subsets.
- Hybrid hierarchical + modular nonoverlapping (${{D}_5}$): one column affects all traits; the other two split the remaining traits into disjoint subsets.

These maps are held fixed within a scenario and all the topologies are completely defined in the supplementary methods document.

### ${\boldsymbol{Z\ }}$ variance regimes

In this section we define three latent-variance regimes for the diagonal matrix *Z*, which control whether process variances are equal, dominated by one process, or ordered at three distinct levels. $Z\ $is a diagonal matrix. We use three families of latent-variance patterns:

- Homogeneous (${{Z}_1}$): ${{Z}_{jj}}{\mathrm{}} = {\mathrm{}}1$ for all *j* (equal variance across processes).
- One dominant (${{Z}_2}$): one process has variance 1; the other share a smaller common variance (0.5). The dominant process can be any of the development latent.
- Stepwise (${{Z}_3}$): three distinct variance levels (1, 0.5, 0.25) assigned to the processes in all six orderings, so that each process can be highest, middle, or lowest variance depending on the label.

Together, $Z\ $defines the variance allocation regime among developmental processes for a given scenario. All $Z\ $matrices are defined in the Supplementary Methods document.

### Scenarios: which latent process is perturbed

In this section we show that base scenarios are specified by three things: a chosen map ${{D}_a}$, a chosen latent-variance pattern ${{Z}_b}$, and an index $j^{\star} \in\{ {1,...,k} \}$ identifying the target developmental process (the column of $D\ $and the diagonal entry of $Z\ $that will be perturbed). The scenario library (Supplementary Methods) enumerates many such triples (e.g., different combinations of hierarchical vs modular columns and dominant vs minor processes).

In principle we could perturb the system either by changing the target variance ${{Z}_{{{j}^ \star }{{j}^ \star }}}$ or by changing the target column ${{D}_{ \cdot {{j}^ \star }}}$. We chose to perturb only $Z\ $, as the resulting trait covariance $S = DZD^{\top}$ would be identical at every $f\ $under the paired rule for $D\ $(scale ${{D}_{ \cdot {{j}^*}}}$ by $\sqrt f $ while holding $Z\ $fixed). This happens because, with diagonal $Z\ $, each ${{S}_{ab}}$ is a sum over latent columns, and the only term that involves both the ${{j}^ \star }$-th column of $D\ $and ${{Z}_{{{j}^*}{{j}^*}}}$ is multiplied by$f\ $either when ${{Z}_{{{j}^*}{{j}^*}}}$is multiplied by $f\ $or when both ${{D}_{a{{j}^ \star }}}$ and ${{D}_{b{{j}^ \star }}}$ are multiplied by $\sqrt f $. All summaries that depend only on $S\ $are therefore the same; we do not distinguish the two implementations elsewhere.

For the perturbation, we multiply the baseline variance ${{Z}_{{{j}^ \star }{{j}^ \star }}}$ by a scalar $f\in\{ {0.5,\,0.6,...,1.5} \}$, with all other parameters unchanged and$f = 1\ $as the reference.

### Metric 1: eigenvalue standard deviation (${{{\boldsymbol{\sigma }}}_{\boldsymbol{\lambda }}}$)

The eigenvalues of a covariance matrix describe how total trait variance is split across orthogonal principal directions. Calculating the variation of these eigenvalues, therefore, allow us to say how even or uneven is the distribution of the total variance among those axes, if one direction dominates, eigenvalues are very spread out; if variance is spread evenly, they are similar. We then use the standard deviation of the eigenvalues of $S\ $(${{\sigma }_\lambda }$, (Pavlicev et al. 2009)), as an index of spectral inequality, how strongly variance is concentrated in a few leading modes versus distributed across many.

Let ${{\lambda }_1} \ge .\ .\ . \ge {{\lambda }_p}$ be the eigenvalues of *S*. We use the sample standard deviation of those eigenvalues as an integration metric, ${{\sigma }_\lambda }{\mathrm{\ }} = {\mathrm{\ sd}}( {{{\lambda }_1},.\ .\ .,\ {{\lambda }_p}{\mathrm{\ }}} )$. As ${{\sigma }_\lambda }$ increases, eigenvalues spread around their mean increases, which typically means that more variance gets concentrated in fewer leading eigen-directions. Biologically, this is interpreted as increased integration among traits, i.e., traits would co-vary more with each other.

### Metric 2: effective dimensionality (${{{\boldsymbol{k}}}_{{\mathrm{eff}}}}$)

Complementary to ${{\sigma }_\lambda }$, we summarize how many orthogonal directions effectively participate in the variance partition using ${{k}_{{\mathrm{eff}}}}$ (effective dimensionality from the entropy of normalized eigenvalues, O’Keefe et al. 2022). Whereas ${{\sigma }_\lambda }$ measures how unequal the eigenvalues are, ${{k}_{{\mathrm{eff}}}}$ measures how many components are needed to describe the relative allocation of variance, an “effective dimension” between 1 and *p*.

To calculate ${{k}_{{\mathrm{eff}}}}$ we normalize the positive eigenvalues to proportions $q_{\mathcal{i}}= {{\lambda }_{\mathcal{i}}}\Big/{\sum\limits_{\mathrm{\ell }} {\lambda }_{\mathcal{i}}}$ and compute the exponential Shannon entropy of this distribution:


\begin{eqnarray*}
{{k}_{{\mathrm{eff}}}} = \ \exp \left( { - \mathop \sum \limits_i {{q}_i}\log {{q}_i}} \right),
\end{eqnarray*}

with the convention that only positive eigenvalues (above a small numerical tolerance) enter the sum. This is a standard “effective number of components”: if all variance were concentrated on one eigen-direction, ${{k}_{{\mathrm{eff}}}}$ is near 1; if variance were spread evenly across all directions, ${{k}_{{\mathrm{eff}}}}$ approaches *p*. ${{k}_{{\mathrm{eff}}}}$ can be interpreted as the effective number of orthogonal directions in which trait variance is spread after normalizing the spectrum, values near one mean almost all covariance sits in a single dominant axis (strong shared structure among traits), while larger values mean variance is distributed across more independent directions. In short, it summarizes how concentrated versus distributed the joint variation is across the trait space.

### Metric 3: subspace distance (${{{\boldsymbol{d}}}_{{\mathrm{sub}}}}$)

One aspect of covariance structure that spectral summaries like ${{\sigma }_\lambda }$ and ${{k}_{{\mathrm{eff}}}}$ miss is where in trait space the leading variance lies, eigenvalues say how much variance falls along each principal direction, but not which traits define those directions, which is given by eigenvectors. Perturbations can therefore alter associations among traits even when the eigenvalue spectrum looks similar. We summarize eigenvector reorientation with ${{d}_{{\mathrm{sub}}}}$, the distance between the leading principal subspaces of the perturbed and reference *S*, as a complement to ${{\sigma }_\lambda }$ and ${{k}_{{\mathrm{eff}}}}$ (Björck and Golub 1973; Stewart and Sun 1990).

Let ${{S}^{{\mathrm{ref}}}}$ be the covariance at $f = 1$ for the same $( {{{D}^{( 0 )}},\ {{Z}^{( 0 )}}} )$ and target ${{j}^ \star }$, and let $S( f )$ be the covariance at perturbation *f*. Let ${{U}^{{\mathrm{ref}}}}$ and $U( f )$ be orthogonal matrices of eigenvectors of ${{S}^{{\mathrm{ref}}}}$ and $S( f )$ (columns ordered by decreasing eigenvalue). For a fixed rank *r*, let $U_{1:r}^{{\mathrm{ref}}}$ and $U{{( f )}_{1:r}}$ denote the first *r* columns (the leading *r*-dimensional principal subspaces).

Let ${{P}^{{\mathrm{ref}}}} = \ U_{1:r}^{{\mathrm{ref}}}U_{1:r}^{{\mathrm{re}}{{{\mathrm{f}}}^{\top}}}$ and $P( f ) = \ U{{( f )}_{1:r}}U( f )_{1:r}^{\top}$ be the orthogonal projectors onto those subspaces. We define the subspace distance as the Frobenius norm of the difference of the two projectors (Baksalary and Trenkler 2014):


\begin{eqnarray*}
{{d}_{{\mathrm{sub}}}} = ||P\left( f \right) - {{P}^{{\mathrm{ref}}}}\ ||_F = \ \sqrt {\mathop \sum \limits_{a,b} {{{\left( {P{{{\left( f \right)}}_{ab}} - P_{ab}^{{\mathrm{ref}}}} \right)}}^2}}
\end{eqnarray*}

Equivalently, this quantity can be derived from the principal angles between the two subspaces (singular values of $U_{1:r}^{{\mathrm{re}}{{{\mathrm{f}}}^{\top}}}U{{( f )}_{1:r}}$); the implementation computes those angles and then ${{d}_{{\mathrm{sub}}}}$ from the projectors. By construction ${{d}_{{\mathrm{sub}}}}( 1 ) = 0$.

For our analyzes, we fix $p = 6$, for $k = 3\ $we fix $r = 2$ and for $k = 4$ we fix $r = 3$. These comparisons allow us to effectively compare the subspace spanned by either the first two or three principal components (the two or three largest eigenvalues of *S*) between $S( f )$ and ${{S}^{{\mathrm{ref}}}}$, respectively. Thus ${{d}_{{\mathrm{sub}}}}$ measures how much those leading subspace moves under the perturbation.

## Results

Here, we show the results for all the 54 $k = 3$ scenarios while the results for the 60 $k = 4$ can be seen in Supplemental Figs. 3 and 6. All the scenarios can be seen in Supplementary File SY. We chose to present only the $k = 3$ results as the general patterns obtained are the same regardless of the choice for *k*. Each scenario fixes a *D* topology, a *Z* variance allocation across latents, and a target latent index *j*, then applies a controlled perturbation to that target process and compares the resulting trait covariance structure to the reference.

### Integration metrics (${{\sigma }_\lambda }$ and ${{k}_{{\mathrm{eff}}}}$)

**Fig. 3:**
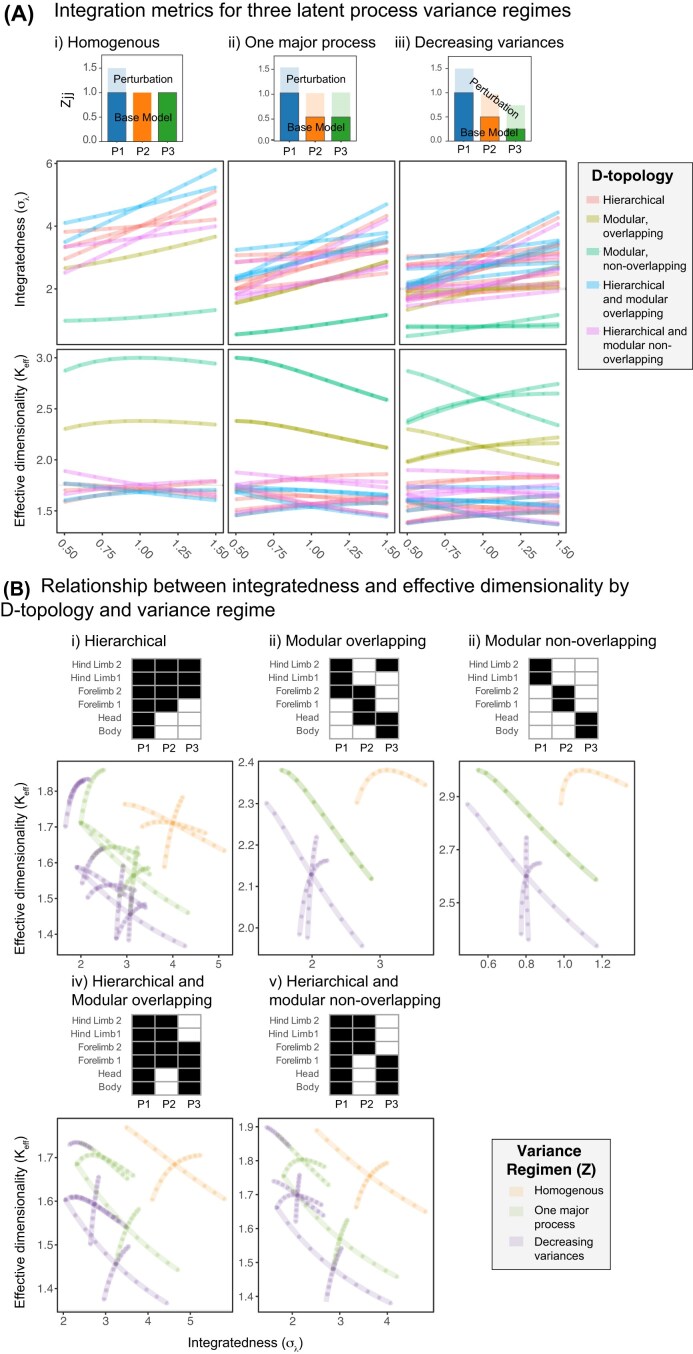
Integration metrics and the relationship between *σ_λ_* and *k_eff_* across variance regimes and *D* topologies (*k = 3*). (A) Trajectories of the two eigenvalue-based summaries as a single latent process variance is perturbed while holding the process-trait map fixed. Columns show three latent variance regimes (homogeneous, one major process, and decreasing variances); rows show *σ_λ_* (eigenvalue dispersion) and *k_eff_* (effective dimensionality of the normalized spectrum). Each colored line is one scenario; colors denote *D* topology class (hierarchical, modular overlapping, modular nonoverlapping, and two hybrid maps). Inset barplots show the reference and perturbed latent variances for the three processes. (B) Relationship between *σ_λ_* and *k_eff_* within each *D* topology, with panels grouped by topology and points/paths colored by variance regime. Each curve traces a single scenario through the (*σ_λ_, k_eff_*) plane across the perturbation trajectory, illustrating distinct response types (negative versus positive association) that depend on both variance regime and topology.

In Fig. 3A, we summarize how the two eigenvalue-based integration metrics (${{\sigma }_\lambda }$ and ${{k}_{{\mathrm{eff}}}}$) respond to the same one-parameter perturbation of a single latent variance component (x-axis, perturbation *f*), while holding the *D* topology fixed within each scenario. For $k\ = \ 3$, ${{\sigma }_\lambda }$ typically increases across the perturbation trajectory, whereas ${{k}_{{\mathrm{eff}}}}$ often decreases or changes more weakly; differences among *Z* variance regimes modulate the magnitude of these shifts but do not remove the overall contrast. The *D* topology sets the baseline organization of these responses: in hierarchical and mixed (hierarchical plus modular) maps, multiple latent processes act on shared trait combinations, which tends to concentrate variance into one or a few dominant directions, leading to higher ${{\sigma }_\lambda }$ and lower ${{k}_{{\mathrm{eff}}}}$. In modular maps, processes act on more separated trait combinations, so variance is distributed across more orthogonal directions, which tends to lower ${{\sigma }_\lambda }$ and keep ${{k}_{{\mathrm{eff}}}}$ higher.

Comparing to $k = 4$, the qualitative story stays the same but the separation by topology becomes more pronounced and the range of responses expands, especially for ${{k}_{{\mathrm{eff}}}}$ (Supplemental Fig. 1a). With four latent contributors, we still see hierarchical/hybrid trajectories anchored to low ${{k}_{{\mathrm{eff}}}}$ and high ${{\sigma }_\lambda }$​, while modular trajectories sit much higher in ${{k}_{{\mathrm{eff}}}}$ and lower in ${{\sigma }_\lambda }$; the additional latent channel effectively provides an extra degree of freedom that can either reinforce a shared axis (in overlap-heavy maps) or populate additional directions (in modular maps), thereby widening the observable contrast. In that sense, increasing *k* does not change the interpretation of the two integration metrics, but it does make their joint response surface sharper, the same perturbation protocol reveals more clearly how topology and variance allocation jointly shape spectral concentration (${{\sigma }_\lambda }$) versus effective spread across directions (${{k}_{{\mathrm{eff}}}}$).

### Relationship between ${{\sigma }_\lambda }$ and ${{k}_{{\mathrm{eff}}}}$

In Fig. 3b ($k = 3$) and Fig. S1b ($k = 4$), we display each scenario as a trajectory in the (${{\sigma }_\lambda }$, ${{k}_{{\mathrm{eff}}}}$) plane as the perturbation factor *f* varies, with panels faceted by *D* topology and trajectories colored by *Z* variance regime. To summarize the overall tendency of each trajectory, we compute the Pearson correlation between ${{\sigma }_\lambda }$and ${{k}_{{\mathrm{eff}}}}$ across the sampled *f* values within each scenario. For $k = 3$, this yields 26/54 scenarios with positive correlations and 28/54 with negative correlations; for $k = 4$, this yields 28/60 positive and 32/60 negative scenarios. We therefore observe two common relationship types: scenarios in which ${{\sigma }_\lambda }$ and ${{k}_{{\mathrm{eff}}}}$ tend to increase or decrease together (positive association), and scenarios in which increases in ${{\sigma }_\lambda }$ tend to coincide with decreases in ${{k}_{{\mathrm{eff}}}}$ (negative association).

The sign of the ${{\sigma }_\lambda }$—${{k}_{{\mathrm{eff}}}}$ association depends on what kind of latent process is being perturbed, and the same qualitative pattern is present for $k\ = \ 3$ and$\ k\ = \ 4$. When the perturbed process is a major variance contributor and is broadly connected to traits in the map, *σ_λ_* and ${{k}_{{\mathrm{eff}}}}$ are usually negatively related: changing that process tends to concentrate variance into one or a few dominant trait-space directions (${{\sigma }_\lambda }$ increases while ${{k}_{{\mathrm{eff}}}}$ decreases). When the perturbed process is lower-variance and/or more restricted in how it reaches traits, the association is more often positive: changing it tends to add variance in additional directions without collapsing the spectrum onto the leading mode (${{k}_{{\mathrm{eff}}}}$ stays high or increases as ${{\sigma }_\lambda }$ changes). This also explains why apparent “exceptions” appear if one considers variance rank alone. In both *k* settings, the few cases where a major-variance perturbation shows a positive relationship occur when that major contributor is structurally constrained by the map (for example, it affects a restricted trait subset or occurs in a modular nonoverlapping configuration), limiting its ability to dominate the global spectrum. Taken together, this plot complements the univariate trajectories by showing not only how each summary changes under perturbation, but also how their covariation reflects both the variance rank of the perturbed process and its inclusiveness versus restriction in the map.

### Subspace distance

**Fig. 4:**
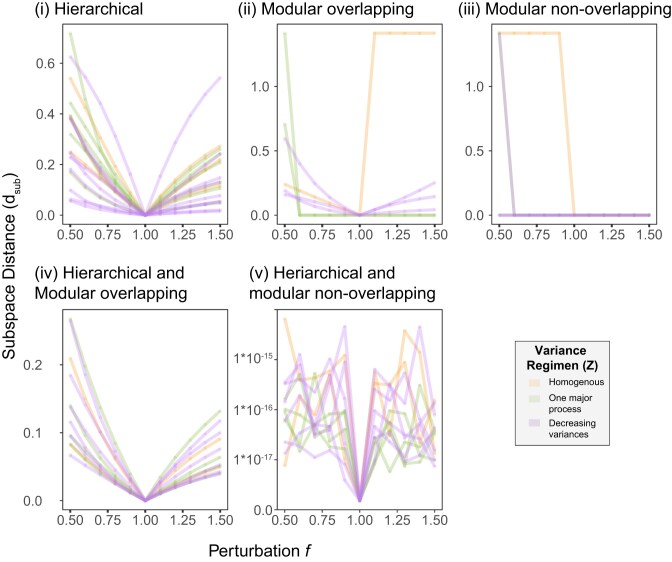
Subspace reorientation distance *d_sub_* as a function of the perturbation *f*, across *D* topologies and *Z* variance regimes (*k = 3*). Each panel corresponds to a process–trait map class (hierarchical; modular overlapping; modular nonoverlapping; and two hybrid maps), and each colored trajectory is one scenario in which a single latent process variance is perturbed while the map is held fixed. By construction, all trajectories pass through *d*_sub_ = 0 at *f* = 1 (the reference covariance). Across scenarios, *d*_sub_ often remains near zero over a wide portion of the perturbation range, indicating little rotation of the leading principal subspace relative to the reference, but some scenarios show abrupt increases, consistent with regimes where the dominant trait-space directions reorganize. Colors indicate the *Z* variance regime (homogeneous variances, one major process, or decreasing variances).

In this section, we quantify how strongly the perturbation changes the dominant covariance structure by measuring the subspace distance ${{d}_{{\mathrm{sub}}}}( f )$ between the leading principal subspace of each perturbed covariance matrix and that of the reference. In Figs. 4 ($k = 3$) and S2 ($k = 4$), each line shows one scenario across the perturbation grid, with panels faceted by *D* topology and color indicating the *Z* variance regime. By construction, all trajectories pass through ${{d}_{{\mathrm{sub}}}}( 1 ) = 0$. Across both *k* settings, we observe that many trajectories remain close to zero over large portions of the perturbation range, whereas a smaller subset shows pronounced departures from zero, including abrupt step-like increases in some scenarios. Thus, subspace reorientation is not a uniform consequence of changing a single latent variance component; instead, the extent and shape of ${{d}_{{\mathrm{sub}}}}( f )$ trajectories depend strongly on scenario structure.

The dependence on *D* topology is visually prominent. In $k = 3$, nonoverlapping modular scenarios show ${{d}_{{\mathrm{sub}}}}( f )$ values that are essentially at or near zero for most trajectories across most of the *f* range, whereas hierarchical and hierarchical-overlapping mixtures more often show smooth, graded departures from zero as *f* moves away from 1. Overlapping modular scenarios contain both near-zero trajectories and a few scenarios with sharp increases, where ${{d}_{{\mathrm{sub}}}}( f )$ changes little near $f = 1$ and then jumps to much larger values. In $k = 4$, the same qualitative partition remains: hierarchical panels show many smooth, V-shaped trajectories around $f = 1$, while modular panels again include many near-zero paths but also include conspicuous discontinuous changes in ${{d}_{{\mathrm{sub}}}}( f )$ for a subset of scenarios.

**Fig. 5:**
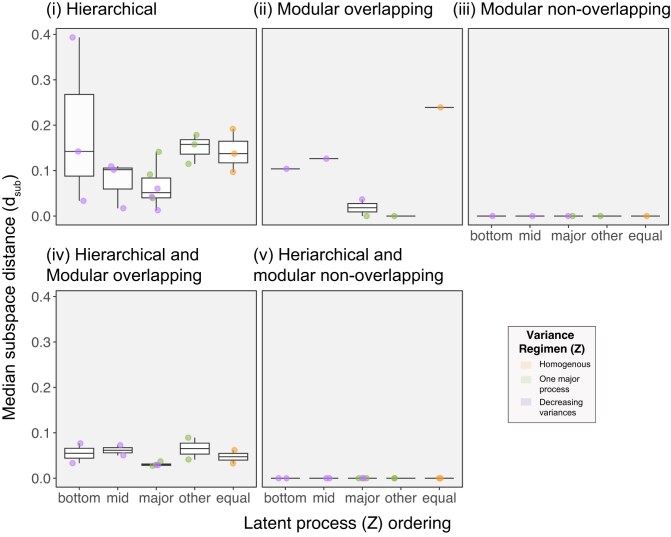
Median subspace reorientation across scenarios, by perturbed-process role and *D* topology (*k = 3*). For each scenario, we summarize the reorientation trajectory *d*_sub_ over the perturbation range by its median value (one point per scenario). The x-axis groups scenarios by the role of the perturbed latent process in the *Z* variance hierarchy; panels correspond to *D* topology class. Boxplots summarize the distribution of median *d*_sub_ across scenarios within each role, and jittered points show individual scenarios; point color indicates the *Z* variance regime (homogeneous, one major process, or decreasing variances). Low medians indicate that the leading principal subspace remains close to the reference across the perturbation range, whereas higher medians indicate stronger and more sustained reorientation.

Fig. 5 ($k = 3$) and S4 ($k = 4$) summarize these reorientation trajectories by the median of ${{d}_{{\mathrm{sub}}}}( f )$ across perturbation values, plotting one point per scenario and grouping scenarios by the role of the perturbed latent within the *Z* variance hierarchy (x-axis), with panels again faceted by *D* topology. This summary reinforces two features visible in Fig. 4. First, several topology classes concentrate near median ${{d}_{{\mathrm{sub}}}} \approx 0$ across many *Z*-roles, indicating that substantial reorientation is not typical for all scenarios. Second, the scenarios with larger median reorientation are concentrated in specific topology-role combinations, especially scenarios involving non-major *Z* contributors. Moving from $k = 3$ (where ${{d}_{{\mathrm{sub}}}}$ compares the leading 2D subspace) to $k = 4$ (where ${{d}_{{\mathrm{sub}}}}$ compares the leading 3D subspace) preserves the overall picture while allowing larger median distances and more extreme outliers in some modular panels, consistent with the broader set of leading directions being tracked when *k* increases.

### Three metrics together

In Fig. S5 ($k = 3$) and S6 ($k = 4$), we summarize each scenario as a path traced in a three-dimensional space of covariance summaries as we vary the perturbation factor *f*. Each trajectory, therefore, represents how a single covariance matrix *S* moves jointly in (${{\sigma }_\lambda }$, ${{k}_{{\mathrm{eff}}}}$, ${{d}_{{\mathrm{sub}}}}$) as we move away from the reference point at $f = 1$. We can observe that trajectories do not fill the space uniformly, forming a structured cloud with two prominent regions and a comparatively sparse interior instead. One extreme of this volume contains paths with relatively larger ${{\sigma }_\lambda }$ and smaller ${{k}_{{\mathrm{eff}}}}$, while the other contains paths with smaller ${{\sigma }_\lambda }$ and larger ${{k}_{{\mathrm{eff}}}}$. In both *k* settings, this organization indicates a strong trade-off between spectral inequality and effective dimensionality: as trajectories move toward higher ${{\sigma }_\lambda }$, they tend to sit in lower ranges of ${{k}_{{\mathrm{eff}}}}$, and the region where both ${{\sigma }_\lambda }$ and ${{k}_{{\mathrm{eff}}}}$ are simultaneously large is rarely occupied by our perturbation paths.

The third axis, ${{d}_{{\mathrm{sub}}}}$, adds a distinct pattern that is not reducible to the two eigenvalue-based summaries. In both Figs. S5 and Fig. S6, we observe many trajectories that remain relatively close to the reference orientation over part of the perturbation range (low ${{d}_{{\mathrm{sub}}}}$), alongside a subset of trajectories that show pronounced excursions in ${{d}_{{\mathrm{sub}}}}$ that appear as near-vertical movements in the snapshot view (large changes in ${{d}_{{\mathrm{sub}}}}$ with comparatively modest movement in ${{\sigma }_\lambda }$ and ${{k}_{{\mathrm{eff}}}}$ at that moment). Figs. S5 and S6 therefore separate trajectories that primarily vary by eigenvalue structure from trajectories that include strong reorientation of the leading covariance subspace, even when their spectral summaries remain in similar ranges.

Comparing Fig. S5 ($k = 3$) to S6 ($k = 4$), the overall geometry of the ensemble is preserved under the same perturbation protocol: trajectories remain organized along the same broad ${{\sigma }_\lambda }$-${{k}_{{\mathrm{eff}}}}$ trade-off, and ${{d}_{{\mathrm{sub}}}}$ continues to separate scenarios with small versus large reorientation. At the same time, Fig. S6 shows trajectories extending over a wider range of ${{k}_{{\mathrm{eff}}}}$ values, and it includes several pronounced ${{d}_{{\mathrm{sub}}}}$ excursions that stand out as tall vertical segments. Thus, adding an additional latent developmental process (and comparing a higher-rank leading subspace in $k = 4$) preserves the qualitative organization seen at $k = 3$ while expanding the range of joint responses that are visible when we view the three summaries together.

### Metrics sensitivity: Is changes in Variance or *D* topology more important?

**Fig. 6:**
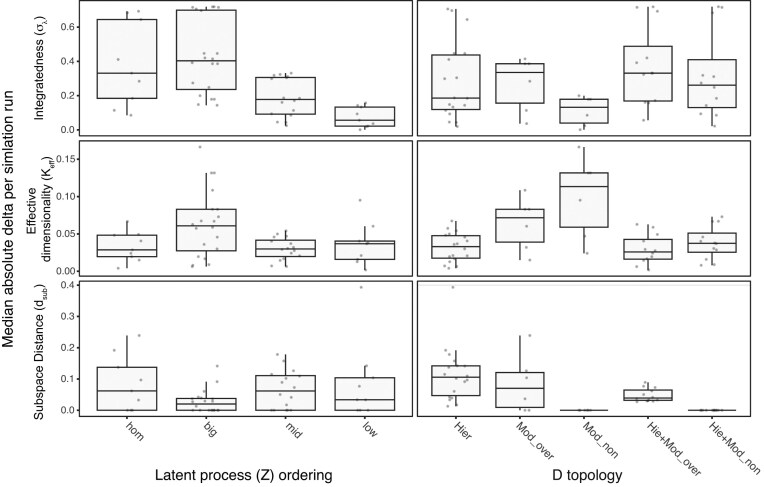
Coarse sensitivity summaries for the three metrics, grouped by perturbed-process role and by *D* topology (*k = 3*). For each scenario, we summarize the perturbation trajectory by a single sensitivity statistic: the median absolute change between neighboring perturbation steps, computed separately for *σ_λ_, k*_eff_, and *d*_sub_ (rows). Left column groups scenarios by the role of the perturbed latent process in the *Z* hierarchy; right column groups the same scenario summaries by *D* topology class. Boxplots show the distribution of per-scenario sensitivities within each group and points show individual scenarios. Across groups, the distributions overlap strongly and remain on similar scales, indicating that sensitivity is not driven by variance organization alone or by topology alone, but depends on the specific scenario (the combination of map structure, variance regime, and which process is perturbed).

To check whether changes in variance or in topology have greater effects on how the three metrics respond to perturbations we gathered per run summaries and plotted these distributions according to, first their *Z*-roles and then according to the topologies in which they were found (Fig. 6). For each run, we recorded the median value of all the absolute differences among neighboring perturbation steps and this was done for the three metrics. The overlapping ranges in Fig. 6 make it hard to claim that either changes in the variance pattern or in the process–trait map systematically produces larger step-to-step movement in ${{\sigma }_\lambda }$, ${{k}_{{\mathrm{eff}}}}$, or ${{d}_{{\mathrm{sub}}}}$. What looks “more sensitive” depends on the particular scenario—how the map is organized, how much variance is allocated, and which process is being perturbed—rather than a clear, overall winner between the two factors.

## DISCUSSION

The concept of integration has deep and diverse origins in evolutionary biology. For Darwin, correlations among traits were clearly important but also subsumed, in *The Origin of Species*, as an explanation for features that could only have arisen as byproducts of selection on some other trait with which they are correlated (Darwin 1859). A very different origin can be seen in the work of German-speaking embryologists such as Gegenbaur (1878). Here we see the idea that organisms are composed of interrelated parts whose harmonious co-existence and function is a central feature of complex organisms. The tension between these two different but related ideas has persisted through the history of evolutionary biology and into the advent of evolutionary-developmental biology as a coherent program of enquiry. Inspired by Darwin’s “laws of correlated growth,’’ Galton sought to use coordinated variation in two traits to uncover common underlying causes, famously inventing the correlation coefficient as a byproduct (Galton 1889). Following this line, Sewall Wright invented factor analysis to identify underlying patterns in sets of trait correlations, distinguishing general and special size factors for limb and skull traits in hens (Wright 1932). Simpson (1953) built on this idea when searching for coherent definitions of traits in order to quantify evolutionary change. He argued that sets of correlated traits should be less arbitrary things to measure than individual traits because such sets would map on to some form of underlying organization with organisms. This is the idea that set Olson and Miller ( 1951, 1958) on the path to the concept of morphological integration.

But the version of integration implicit in the work of Gegenbaur and others also persisted and evolved (1878). This is even true of adherents of the modern synthesis, including Julian Huxley who coined that term. Huxley’s idea that overall organismal forms are underlying by interacting growth factors and growth centers, for example, speaks to a vision of organisms as integrated wholes composed of interrelated parts (Huxley 1932). But it is perhaps among scholars who did not identify closely with the modern synthesis that this thread is developed most fully. For Goldschmidt, interrelations among organismal parts represented such a powerful constraint on evolution that they could only be overcome by radical reorganization (Goldschmidt 1940). For Waddington, epigenetic interactions within developmental systems resulted in alternative stabilized paths of potential phenotypic variation (1957). These paths, or the grooves in his epigenetic landscape, represent developmental trajectories of whole organisms, implying integrated variation among component parts in some multivariate phenotypic direction (1957).

These two conceptual paths, one representing the Darwin-inspired search for meaningful units of evolutionary change and the other representing a desire to better understand the organizational features of organisms, come together in the modern concept of integration which we will refer to here as the *latent variable model*. This model begins with work performed independently, albeit with correspondence and interaction, by Gunter Wagner and James Cheverud. Drawing on both conceptual origins but also inspired by Lande’s multivariate methods for quantifying the response to selection (1979), Cheverud (1988, 1996, 1982, 1984, 1985, 1988, 1996) and Wagner (1984, 1989) introduced two closely related ideas that transformed the concept of integration. The first is that sets of phenotypic traits have a covariance structure and that the form of this structure is in some way related to the developmental and environmental determinants of multivariate variation. The key insight here is that these relations can be understood through a latent variable structure (Wagner 1984). These latent variables and the manner in which traits load on to them, they argued, are meaningful because they reflect the developmental architecture that shapes multivariate variation, influences the response to selection but is, in turn, also a product of selection acting on functionally related traits (Cheverud 1996). The second idea is that since multivariate variation is shaped by developmental determinants, those determinants can be influenced by both genetic and environmental perturbations (Cheverud 1982). This is why environmental and genetic covariance matrices for complex traits so often align, a phenomenon that Roff dubbed “the Cheverud conjecture” (Roff 1997).

Building on this latent-variable view of integration, the palimpsest framework addresses cases in which many developmental processes contribute to the same trait covariance structure (Hallgrimsson et al. 2009). Its premise is that variation, whether of genetic or environmental origin, flows through developmental processes and that variation in these processes underlies covariation among traits. Since there are many such processes and they overlap in both time and space, the result is a palimpsest in which the covariance effects of different processes interact, overwrite or amplify each other’s effects. When faced with real-world data, the palimpsest model invites a set of questions that can help unravel the causes for observed differences in covariance patterns. For instance, when two samples differ in phenotypic variance for some multivariate set of traits, the palimpsest model invites the question of how this difference in variance is apportioned across covariance-generating processes (the latent processes in our model). Heuristically, when a variance change is not spread equally across processes, the palimpsest picture suggests that how covariance changes will depend on which processes gain or lose influence. For example, amplifying a process that is already a weak contributor can redistribute variation across directions in trait space, whereas amplifying a strong contributor can more easily reinforce an already-dominant axis- or subspace-like organization. If two processes were nearly matched in their contribution, even modest reallocation can reorder which directions dominate, sometimes producing abrupt shifts in the geometry of covariation. Many of these qualitative expectations have been explored in mutant models where the developmental sources of covariance perturbation are at least partially interpretable (Hallgrimsson et al. 2006, 2009, 2020). Ontogenetic covariance series provide a complementary data type because they allow trait covariance to be compared along a developmental trajectory rather than summarized at a single time point. Each interval contributes its own covariance structure, and comparing matrices from one stage to the next makes it possible to ask how much each developmental segment reshapes integration and how those segment-level effects combine to produce the covariance pattern observed in later life. That sequential structure is especially natural for palimpsest thinking: ontogeny becomes an ordered set of intermediate states whose covariance effects accumulate, so that the adult trait covariance can be read as the outcome of overlapping developmental inputs acting in time. Hubbe et al. (2023) applied this logic to postnatal cranial series in marsupials and placentals, comparing trait covariance across ontogenetic stages and showing where along the trajectory integration repatterns most strongly and where covariance structure stabilizes. The value of such data is not only that covariance changes with age, but that the path of change can be decomposed by stage, supporting hypotheses about which developmental processes, active in which intervals, contribute most to the realized covariance structure at maturity.

Environmental perturbation experiments, artificial selection, and phylogenetic comparison extend the same contrast-based logic along other axes. Nutrition, stress, or related factors can be though of as controlled perturbation and manipulated under controlled designs, and their effects of trait covariance then studied. Trait covariance can depend on environment because characters respond through reaction norms that need not be parallel across traits (Gebhardt and Stearns 1993; Via and Lande 1985; Matesanz et al. 2021; Handelsman 2014 #62,832) cranial morphometric work under factorial nutritional stress illustrates this across ontogeny and among cranial modules (Gonzalez et al. 2011a, 2011b), as does multivariate evidence that plasticity can shift major axes of variation and trait coordination (Lind et al. 2015; Stoks et al. 2016; Reger et al. 2018). Palimpsest thinking organizes treatment by ontogeny contrasts (timing, dose, duration) and asks how covariation responds along that contrast set. Artificial selection provides a generational contrast on a known selective axis, comparing trait covariance among selected and control lines across generations and at endpoint and relating multivariate responses to developmental processes shared among directly and indirectly affected traits (Marchini et al. 2014; Marchini and Rolian 2018; Unger et al. 2021). Phylogenetic comparison of integration and modularity yields a library of covariance structures among lineages in broad samples (Marroig et al. 2009; Porto et al. 2009; Watanabe et al. 2019), complementing experimental trajectories by asking where coupling among trait complexes has diverged or remained conserved across clades. Across mutants, ontogenetic series, environmental treatments, selection lines, and phylogenetic samples, the palimpsest framework improves interpretation by treating differences in trait covariance as structured contrasts and asking how integration is repatterned along each contrast set: perturbation, developmental time, environment, generation, or lineage.

A further implication of the palimpsest framework is that the process-trait map (topology *D*) is not a passive backdrop. Instead, it directs the ways in which latent process variation is projected into phenotypic covariance, so the same latent-variance change can yield different multivariate outcomes depending on *D*, interacting with which process is perturbed under *Z*. In our controlled simulations we make this routing explicit: we follow covariance responses when latent variances are perturbed while holding a fixed *D*, and we contrast maps that differ in how overlapping versus more separated process-trait organization channels variance into shared versus more partitioned trait combinations. Our simulations illustrate this coupling: responses depend on the joint configuration of variance allocation and routing-by-topology, rather than on a simple global ranking of one factor over the other.

Beyond showing how the palimpsest model is useful as a framework, helping us envision how differences in trait covariance can arise from changes in latent process variances and/or in the map connecting latent processes to traits, it can also be useful as a diagnostic tool for the generative parameters that could have produced a given covariance matrix. The idea is operational rather than parametric in the estimation sense: apply controlled perturbations to *S* (relative to a reference, under an explicit protocol) and track a small set of matrix responses using three complementary summaries (spectral inequality, effective spectral dimensionality, and leading-subspace reorientation). Taken together, these responses do not tell us exactly how much each developmental process varies or exactly which traits each process affects. Instead, they show which broad combinations of process variance and process–trait wiring fit the observed pattern of matrix responses and which can be ruled out. That narrows the range of plausible explanations; it does not identify a unique developmental mechanism from a single covariance matrix alone. Across our analyses, four recurring response signatures make this possible. First, the process–trait map topology *D* leaves a persistent imprint on the eigenvalue-only summaries: hierarchical and hybrid maps tend to occupy higher ${{\sigma }_\lambda }$ and lower ${{k}_{{\mathrm{eff}}}}$ than modular non-overlapping maps. Second, *D* also imprints a mark on reorientation: hierarchical scenarios show smoother, graded ${{d}_{{\mathrm{sub}}}}( f )$, whereas modular scenarios more often show long near-zero stretches punctuated by spikes. Third, ${{\sigma }_\lambda }$ almost never decreases along our perturbations, so decreases (when they occur) are diagnostic rather than generic. Fourth, the relationship between ${{\sigma }_\lambda }$ and ${{k}_{{\mathrm{eff}}}}$ along a perturbation trajectory separates scenarios into distinct response types (positive vs negative association), which covary with whether the perturbed latent behaves like a major/global versus minor/local contributor. Below we explain each pattern and how, taken together, they can be used to rule out broad (*D, Z*) ranges for a perturbed matrix even when *S* alone cannot.

The first pattern is the baseline spectral signature induced by topology. When the process–trait map *D* creates broad overlap among developmental processes (hierarchical and hybrid maps), multiple latent contributions act on shared trait combinations and the resulting covariance matrix *S* is dominated by one or a few principal directions. In this case the eigenvalue spectrum is more unequal, so ${{\sigma }_\lambda }$ tends to be higher and the normalized spectrum is more concentrated, so ${{k}_{{\mathrm{eff}}}}$ tends to be lower. When *D* is modular and non-overlapping, processes act on more separated sets of traits and variance is distributed more evenly across multiple trait-space directions, yielding lower ${{\sigma }_\lambda }$ and higher ${{k}_{{\mathrm{eff}}}}$, with modular-overlapping and hybrid cases typically intermediate. This separation is useful for our aim because it provides a coarse diagnostic of modularity from comparisons among matrices. Although the pair (${{\sigma }_\lambda }$, ${{k}_{{\mathrm{eff}}}}$) cannot identify the full set of latent processes, we find that it can help rule out broad classes of *D*: matrices (or perturbation series) that consistently occupy the high-${{\sigma }_\lambda }$, low-${{k}_{{\mathrm{eff}}}}$ region are difficult to reconcile with a purely nonoverlapping modular organization, whereas matrices that remain comparatively even in their spectra (low ${{\sigma }_\lambda }$, high ${{k}_{{\mathrm{eff}}}}$) are difficult to reconcile with strongly hierarchical coupling. Importantly, this topological imprint persists even when we perturb one latent contributor at a time; the perturbation trajectories remain anchored to the baseline spectral organization imposed by *D*.

Reorientation adds a second, independent diagnostic cue that eigenvalues alone do not provide: it asks whether a perturbation mainly changes the amount of variance along the same dominant trait-space directions or instead changes which trait combinations define those dominant directions. We summarize this with the subspace-distance measure ${{d}_{{\mathrm{sub}}}}$ which compares the leading principal subspace of the perturbed covariance matrix to that of the reference along the perturbation path. Because we compare subspaces rather than individual eigenvectors, which can swap identity, ${{d}_{{\mathrm{sub}}}}$ gives a robust summary of how much the dominant directions of covariation rotate. In our simulations, hierarchical maps more often yield smooth, gradual reorientation as the perturbation moves away from $f = 1$, whereas modular and mixed maps more often show long near-zero stretches punctuated by abrupt jumps, consistent with regimes where dominant directions remain stable until the perturbation crosses a point where which directions dominate is reordered. As a diagnostic, these contrasting trajectory shapes help distinguish covariance changes that are primarily rescalings of an existing structure from changes that reflect genuine reorganization of the dominant axes.

At the same time, there is one aspect of the reorientation that we must be careful, which is that eigenvectors become unstable when leading eigenvalues are close (Zelditch et al. 2006). When the leading eigenvalues of *S* are well separated, the leading subspace is well defined and small perturbations typically produce small rotations. In contrast, when several leading eigenvalues are similar in magnitude, the corresponding eigenspace is weakly identified, and small changes in *S* can produce large apparent rotations within that nearly degenerate space even if the eigenvalues change smoothly. Spikes in ${{d}_{{\mathrm{sub}}}}$ can reflect real turnover in which axes dominate, but they can also arise because we are comparing directions that are not stably defined when the spectrum is flat. Practically, this means that large ${{d}_{{\mathrm{sub}}}}$ values are most interpretable when the leading structure has an effective separation (or effective rank) that supports stable orientation estimates; when near-ties are present, spikes should be read as flags for regimes where small perturbations can reorder dominant directions, rather than as unambiguous evidence of a discrete developmental “switch.”

A third pattern is how ${{k}_{{\mathrm{eff}}}}$ and ${{\sigma }_\lambda }$ correlate along each perturbation trajectory for each scenario. When the relationship is negative, ${{\sigma }_\lambda }$ rises and ${{k}_{{\mathrm{eff}}}}$ falls, variation becomes more concentrated in one or a few dominant directions as perturbation increases. When the relationship is positive, increasing the perturbation tends to broaden the spectrum instead of collapsing it onto a single leading direction, ${{k}_{{\mathrm{eff}}}}$ stays high or increases while ${{\sigma }_\lambda }$ changes in a way that matches that broader, less single-axis outcome. In our simulations, the negative pattern is more typical when the perturbed developmental process is already a large contributor and/or is broadly coupled through the process-trait map, so changing it mostly strengthens directions that already dominate. The positive pattern is more typical when the perturbed process is smaller or more restricted in how it reaches traits, so changing it tends to lift secondary directions rather than further inflate the top axis (including cases where a process looks important in the latent variance picture but the map keeps it from running the whole spectrum). Reading the sign of this relationship is therefore a coarse diagnostic of whether the perturbed channel behaves like a global driver of the covariance pattern or a more local one, alongside what you learn from map-driven baseline levels and from reorientation (${{d}_{{\mathrm{sub}}}}$).

The fourth recurring signature is the typical direction of change in ${{\sigma }_\lambda }$ along a perturbation trajectory. In our setup the process-trait map is fixed and only one latent variance is perturbed at a time, so each step adds or removes variance along a single trait-space direction determined by that process loading pattern. With overlapping maps, increasing a latent variance usually increases variance along directions that already contribute strongly to the leading structure of the reference covariance, which tends to widen the gap among eigenvalues and raise ${{\sigma }_\lambda }$. Decreasing a latent variance often removes variance from trait combinations that remain fed by other processes, so it does not reliably reduce only the largest eigenvalue; ${{\sigma }_\lambda }$ therefore usually increases or stays on the increasing side of the trajectory, and sustained decreases are uncommon.

A decrease in ${{\sigma }_\lambda }$ is informative because it generally requires geometry that the one-at-a-time variance perturbation rarely satisfies under a fixed map. One mechanism is that variance is added primarily along directions that were weak in the reference while the leading eigenvalues change little; this is most plausible for a minor or trait-restricted process whose loading pattern is nearly orthogonal to the subspace that dominates the reference spectrum, and the perturbation must be large enough that the rise in smaller eigenvalues narrows the spread more than any secondary mode grows at the top. Another mechanism is selective reduction of the variance component most aligned with the current leading direction, shrinking the top eigenvalue faster than the others. A third mechanism is a change in the map itself (or coordinated changes in multiple variances), which can redistribute variance across trait combinations rather than moving along a single fixed loading direction. In all cases, ${{\sigma }_\lambda }$ should be read together with ${{k}_{{\mathrm{eff}}}}$: narrowing of the eigenvalue spread should show a coordinated signature in ${{k}_{{\mathrm{eff}}}}$, whereas large rotations need not mirror large changes in ${{\sigma }_\lambda }$.

A natural question raised by our perturbation framework is whether the response of manifest covariance summaries is driven primarily by variance hierarchy (*Z*) or by topology (*D*). When we compare sensitivities across our scenario library, we do not find a consistent ordering in which one of these dimensions universally produces larger responses across all three metrics. Instead, sensitivity distributions overlap substantially when scenarios are grouped marginally by variance role or by topology, indicating that neither *Z* nor *D* dominates in isolation. The interpretation is that sensitivity is inherently interactive in models of the form $S = DZD^{\top}$ scaling one latent variance changes variance along the trait-space direction implied by that latent’s connections in *D*, so the same perturbation magnitude can be amplified or damped depending on how that direction aligns with the dominant covariance geometry established by the full *D*–*Z* configuration. In short, the impact of *D* versus *Z* depends on the specific *D*–*Z* combination and on which latent process within that combination is perturbed.

Here, we have taken a simulations approach to show how the palimpsest framework can guide interpretation of biological covariance patterns. Conceptually, the palimpsest frames trait covariance as something assembled from overlapping developmental inputs whose effects can compound or partly overwrite one another, so shifts in covariance naturally invite questions about how variability is allocated among latent contributors and how those contributors connect to phenotypic traits. In real biological settings, differences in covariance patterns can arise through shifts in variance allocation among processes, through changes in the process-trait mapping, which complicates interpretations of observed differences in covariance structure. Our perturbation exercise does not estimate latent parameters uniquely from a covariance matrix alone, but it does show how comparable perturbations—tracked with complementary summaries—produce repeatable contrasts that narrow broad compatibility ranges for internal organization.

On those grounds the palimpsest framework serves both as an organizing idea for comparative questions, and as a heuristic model for devising hypothetical latent process variance and process to trait mapping configurations. Comparative questions become concrete when tied to structured contrasts among covariance matrices. Comparative work on mammalian skulls shows that integration patterns can remain broadly shared across orders while integration magnitudes vary markedly, with measurable consequences for evolutionary flexibility (Porto et al. 2009; Marroig et al. 2009). That juxtaposition is difficult to interpret from trait covariance alone, but it becomes a concrete comparative program under a palimpsest view: does greater integration magnitude arise mainly from redistribution of variance among latent developmental processes (concentrating variation along fewer dominant directions) or from repatterned process-to-trait coupling, so that the same processes map to traits in a more integrated modular layout? These alternatives are not mutually exclusive, yet they imply different next steps: ontogenetic contrasts, perturbations, or cross-lineage comparisons aimed at whether repatterning is driven by changed process input versus changed wiring. The palimpsest framework does not resolve that question from a single covariance matrix, but it turns a descriptive macroevolutionary pattern into testable, process-level hypotheses that standard integration metrics do not articulate on their own.

Hypothetical latent configurations can be framed similarly at the scale of experimental perturbation. Such models can be used to frame expectations such as what might be expected to happen when a mutation increases the variance of a specific process in addition to altering the phenotypic mean. Changes in trait means can also complicate these scenarios when they are large, which is a topic deserving of a separate analysis. The palimpsest framework can also be used to generate hypotheses that distinguish between the expectations of hypothetical changes in the mapping of processes to traits from changes in the variances of those processes. Finally, the palimpsest framework can serve to ground studies of comparative changes in covariance patterns in terms of the range of interpretations that may be compatible with some observed pattern in nature. Given the importance of trait covariance patterns in the study of the relationship between evolution and development more generally, we believe that these are important contributions.

## Supplementary Material

icag111_Supplemental_Files

## Data Availability

Code is available at https://github.com/Hallgrimsson-lab.

## Notes

From the symposium “Integrating integration: How cellular mechanisms of trait covariation shape evolutionary patterns of phenotypic integration” presented at the annual meeting of the Society for Integrative and Comparative Biology, January 3-7th, 2026.
