# Supplementary material for: Revisiting the Palimpsest: Exploring the Interaction of the Variance and Structure of Developmental Determinants of Phenotypic Covariance
Source: Integr Comp Biol. 2026 Jul 7;66:icag111. doi: 10.1093/icb/icag111 (PMC13446123; doi:10.1093/icb/icag111)
Supplement: Supplementary file 1 [file icag111_supplemental_files.zip › icb-2026-0161-File009.pdf]

Supplementary Information

Supplementary Figures — static panels; Figures S5–S6 show joint variation of σ_λ, k_{eff}, and d_{sub} in three dimensions ($k = 3$ and $k = 4$)

Contents

Figure S1 — Integration metrics along f 2
Figure S2 — Subspace reorientation d_{sub} vs. f 3
Figure S3 — σ_λ versus k_{eff} ($k = 4$) 4
Figure S4 — Marginal sensitivity overview 5
Figure S5 — Joint variation of σ_λ, k_{eff}, and d_{sub} ($k = 3$) 6
Figure S6 — Joint variation of σ_λ, k_{eff}, and d_{sub} ($k = 4$) 7

Overview. Figures S1–S4 summarize integration metrics, subspace reorientation, σ_λ versus k_{eff} in the plane, and marginal sensitivity for the $k = 4$ scenario library; Figures S5 and S6 plot joint trajectories of σ_λ, k_{eff}, and d_{sub} under Z-variance sweeps for the $k = 3$ and $k = 4$ scenario libraries (three-dimensional projections). Panels below are included as vector PDFs scaled to **full text width** so wide multipanel figures are not clipped.

Figure S1 — Integration metrics along f

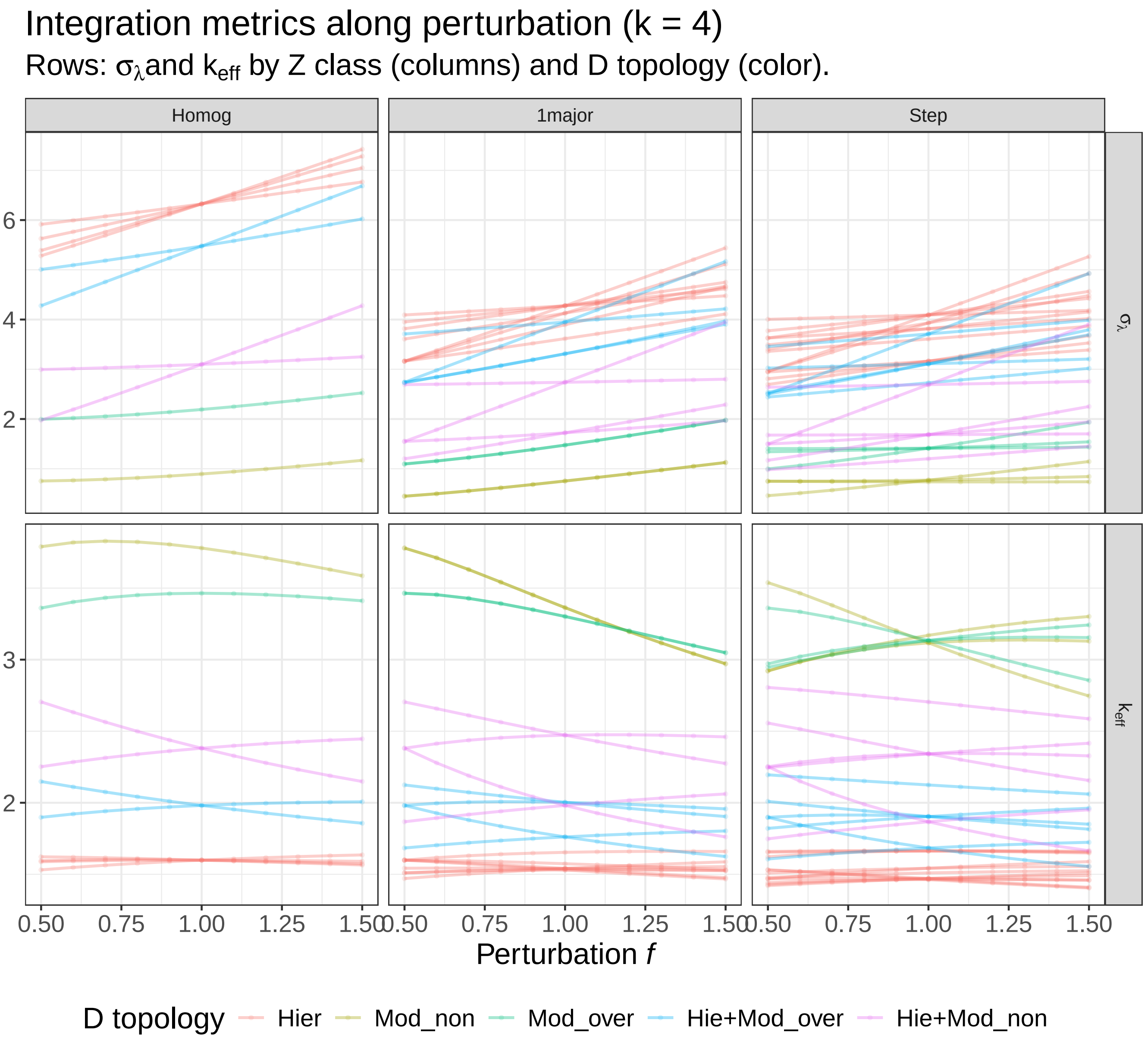

Figure S1: Integration indices as a function of the perturbation multiplier f (four latent processes). **Columns:** Z variance regime — homogeneous (Homog), one dominant process (1major), stepwise variances (Step). **Rows:** σ_λ, standard deviation of eigenvalues of the trait covariance matrix; k_{eff}, entropy-based effective number of eigenvalues. **Line color:** process–trait topology (D) class (Hier, Mod_non, Mod_over, Hie+Mod_over, Hie+Mod_non). Each translucent curve is one scenario in the library; the perturbation rescales the variance of the targeted latent process.

Figure S2 — Subspace reorientation d_{sub} vs. f

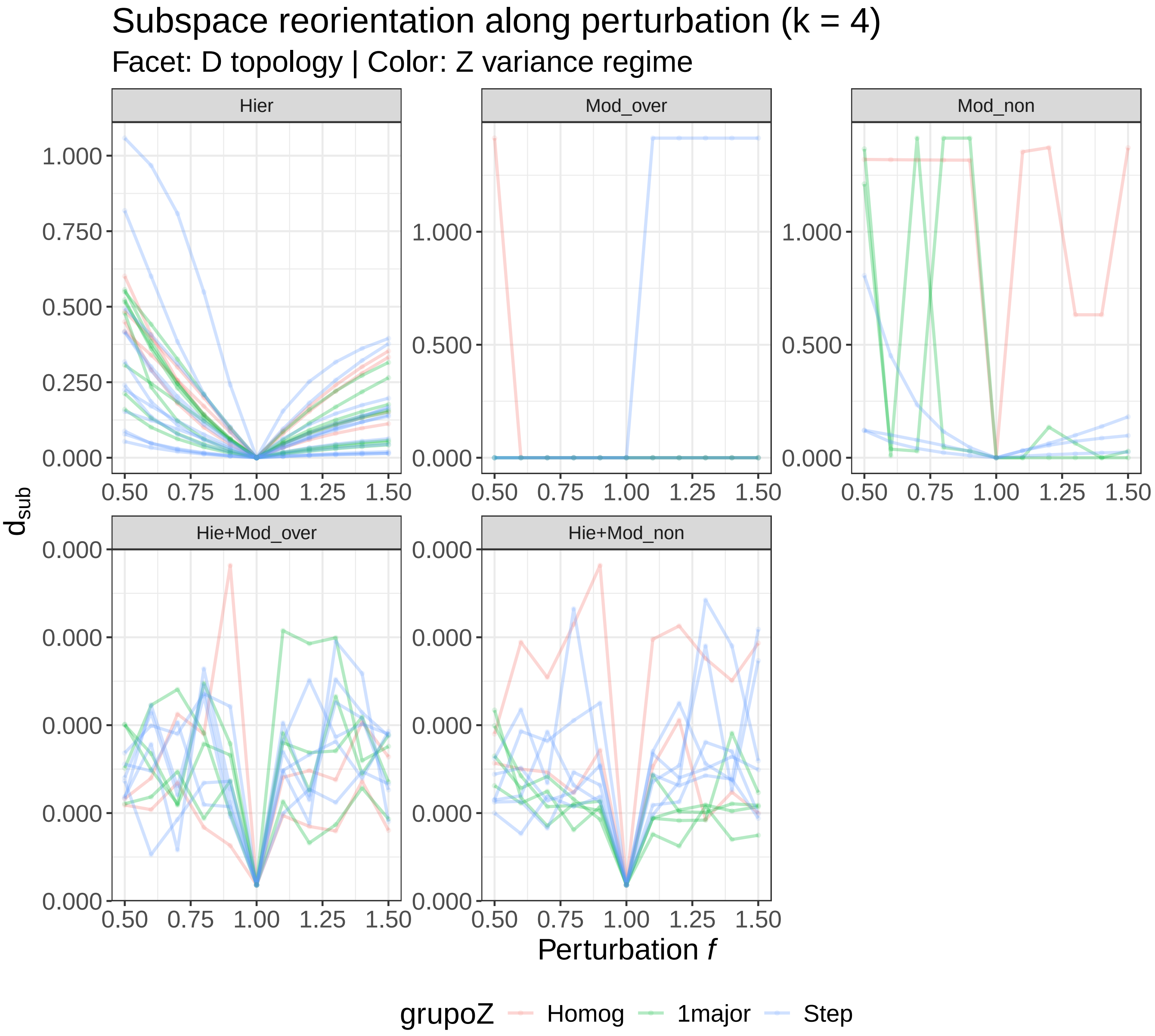

Figure S2: Reorientation distance d_{sub} between the leading $(k-1)$-dimensional principal subspaces of the reference and perturbed trait covariance matrices, plotted against f. **Facets:** D topology class. **Color:** Z variance regime (Homog, 1major, Step). Each faint trajectory corresponds to one scenario; larger d_{sub} indicates stronger rotation of the leading subspace under the latent-variance perturbation.

Figure S3 — σ_λ versus k_{eff} ($k = 4$)

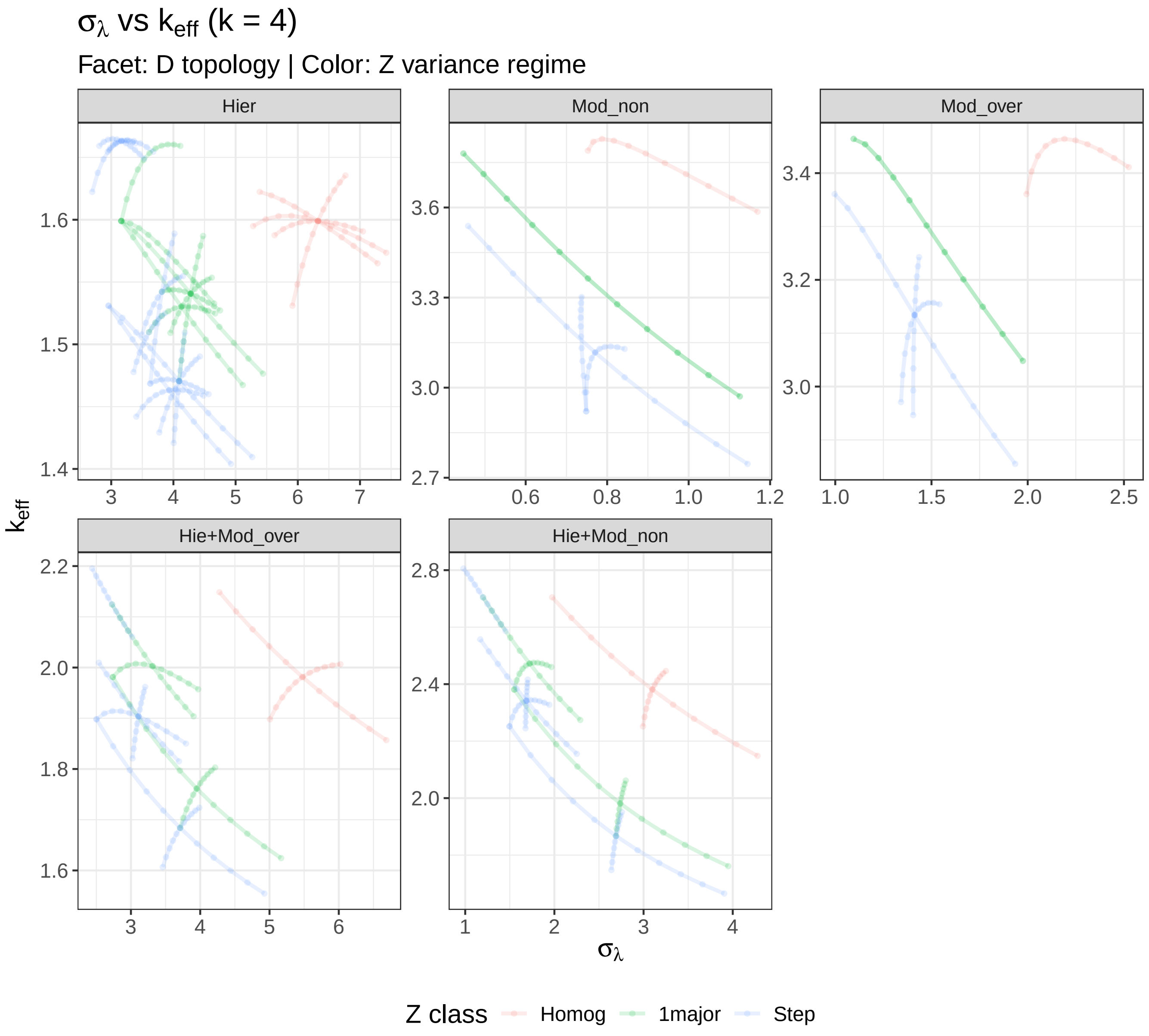

Figure S3: Joint variation of the standard deviation of eigenvalues of the trait covariance matrix (σ_λ) and the entropy-based effective number of eigenvalues (k_{eff}) along Z-variance perturbation sweeps (four latent processes). **Facets:** *D* topology class (Hier, Mod_non, Mod_over, Hie+Mod_over, Hie+Mod_non); facet axes are free to scale. **Color:** Z variance regime (Homog, 1major, Step). Each connected trajectory is one scenario; points and line segments follow increasing perturbation multiplier f within that scenario.

Figure S4 — Marginal sensitivity overview

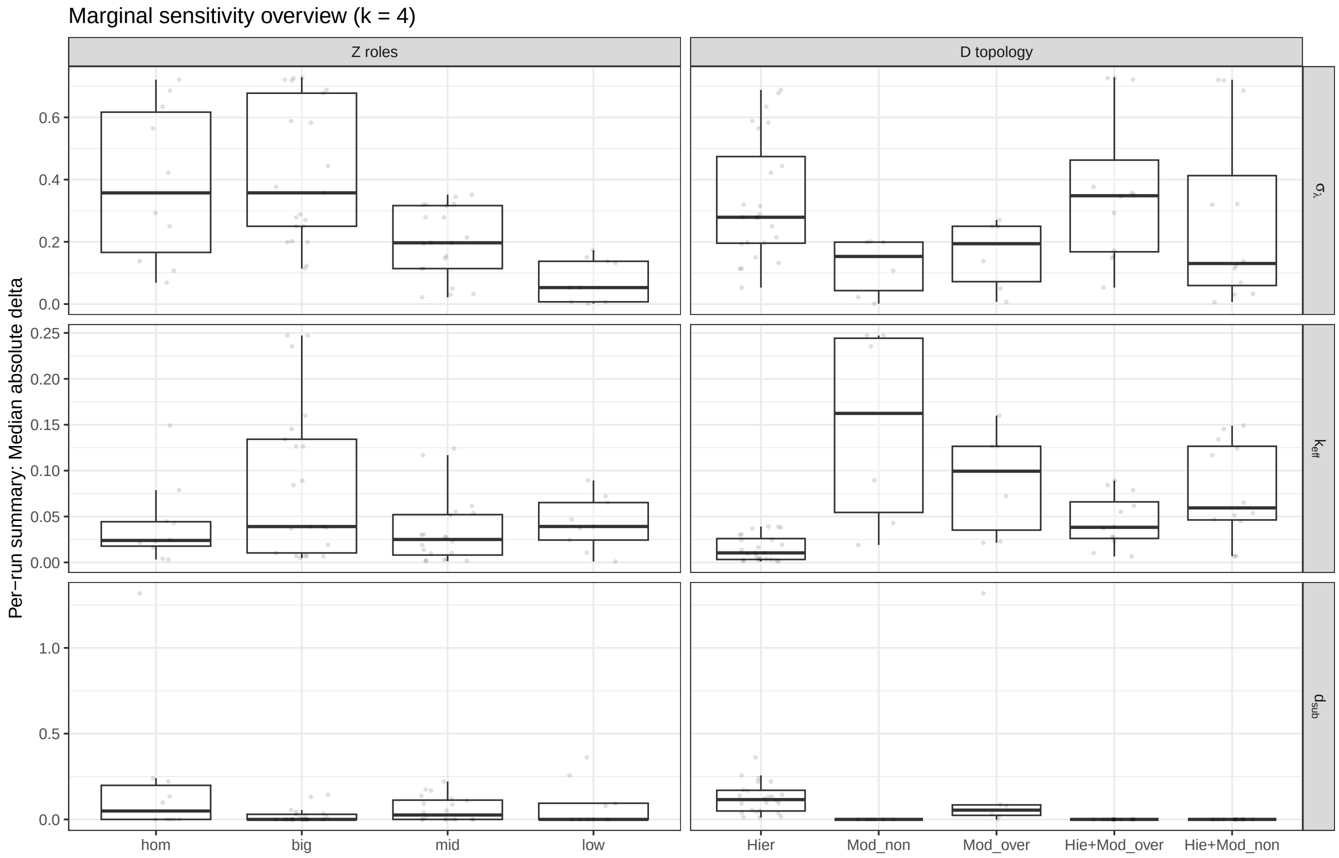

Figure S4: Distribution across scenarios of the median absolute deviation of each metric from its value at the reference lever ($f = 1$), accumulated along the full sweep in f. **Rows:** σ_λ, k_{eff}, and d_{sub}. **Left column:** scenarios grouped by simplified role of the perturbed process under Z. **Right column:** scenarios grouped by D topology label. Boxplots summarize dispersion; jittered points show individual scenarios.

Figure S5 — Joint variation of σ_λ, k_{eff}, and d_{sub} ($k = 3$)

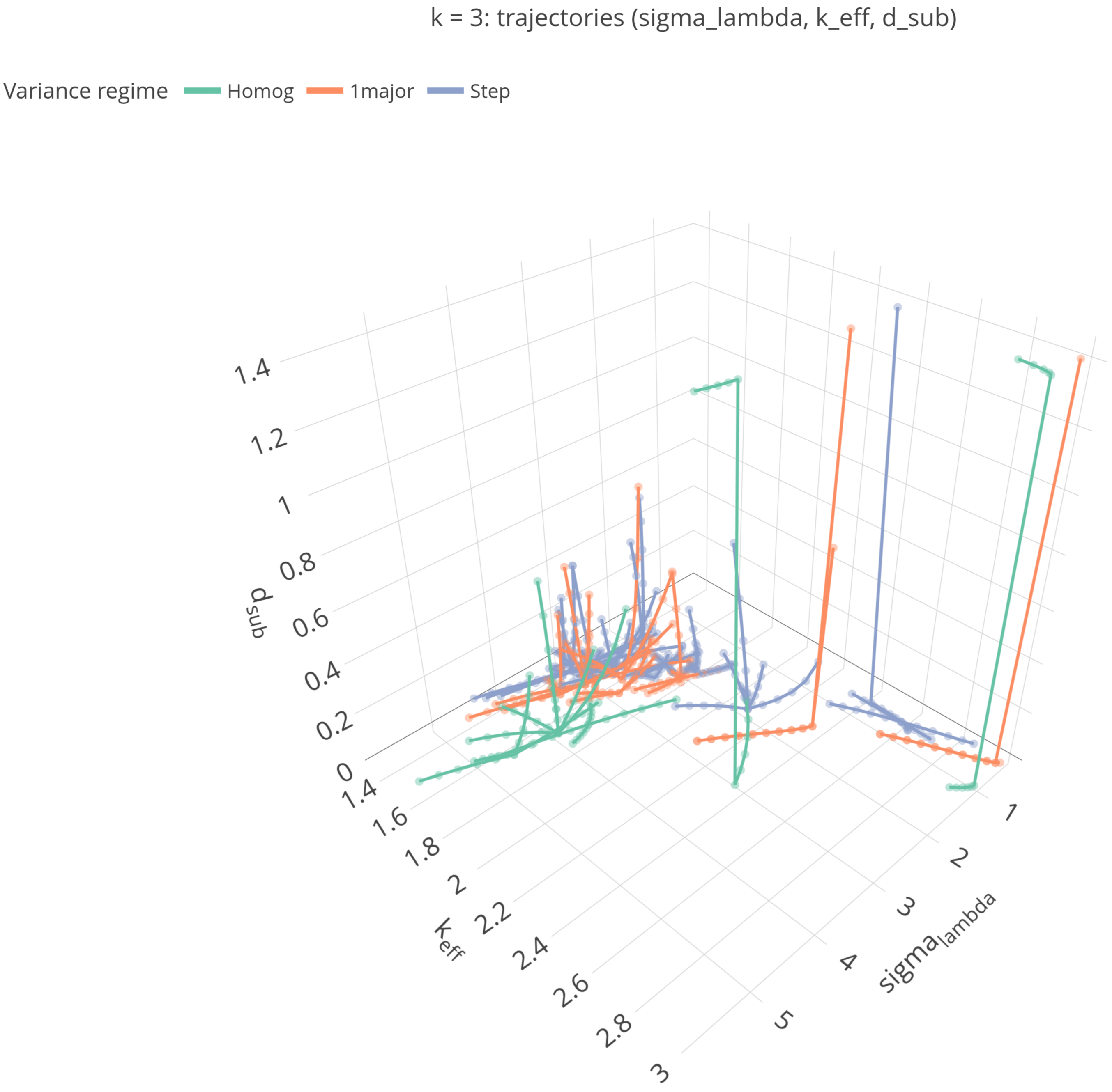

Figure S5: Joint variation of the standard deviation of eigenvalues of the trait covariance matrix (σ_λ), the entropy-based effective number of eigenvalues (k_{eff}), and the subspace reorientation distance (d_{sub}) along Z-variance perturbation sweeps (three latent processes). **Axes:** σ_λ, k_{eff}, and d_{sub} in the three-dimensional plot. **Color:** Z variance regime (Homog, 1major, Step). Each connected trajectory is one scenario; points and line segments follow increasing perturbation multiplier f within that scenario.

Figure S6 — Joint variation of σ_λ, k_{eff}, and d_{sub} ($k = 4$)

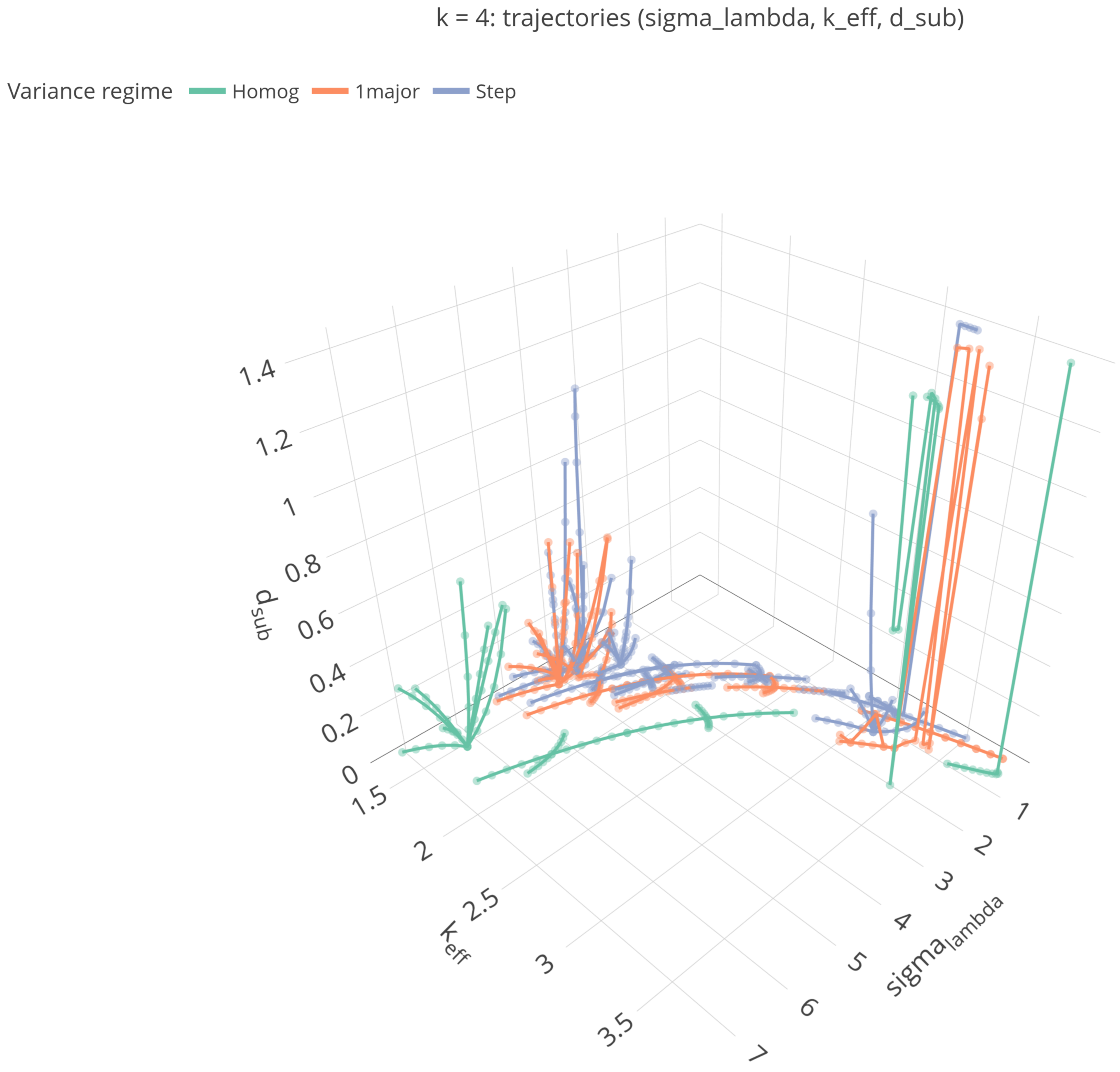

Figure S6: Joint variation of the standard deviation of eigenvalues of the trait covariance matrix (σ_λ), the entropy-based effective number of eigenvalues (k_{eff}), and the subspace reorientation distance (d_{sub}) along Z-variance perturbation sweeps (four latent processes). **Axes:** σ_λ, k_{eff}, and d_{sub} in the three-dimensional plot. **Color:** Z variance regime (Homog, 1major, Step). Each connected trajectory is one scenario; points and line segments follow increasing perturbation multiplier f within that scenario.
