# Supplementary material for: Revisiting the Palimpsest: Exploring the Interaction of the Variance and Structure of Developmental Determinants of Phenotypic Covariance
Source: Integr Comp Biol. 2026 Jul 7;66:icag111. doi: 10.1093/icb/icag111 (PMC13446123; doi:10.1093/icb/icag111)
Supplement: Supplementary file 1 [file icag111_supplemental_files.zip › icb-2026-0161-File010.pdf]

Supplementary File: Scenario library (D, Z, and target process j)

This supplementary file lists the full set of D and Z matrices used in our analyses for $k \in \{3, 4\}$, and it enumerates all scenarios (the combinations of D, Z, and target index j).

Conventions used throughout:

- In each D matrix, rows correspond to traits and columns correspond to latent developmental processes; entries are binary (1 = connection present, 0 = no connection).
- Each Z matrix is diagonal and is fully determined by a vector of latent-process variances; we therefore list each Z as its diagonal variance vector.
- In the scenario tables, j is the target process index (i.e., the index of the latent process that is perturbed).

Scenario library for $k = 3$ (with $p = 6$ traits)

Process–trait maps D

`D1`

1	0	0
1	0	0
1	1	0
1	1	0
1	1	1
1	1	1

`D2`

0	1	1
0	1	1
1	0	1
1	0	1
1	1	0
1	1	0

`D3`

1	0	0
1	0	0
0	1	0
0	1	0
0	0	1
0	0	1

`D4`

1	1	0
1	1	0
1	1	1
1	1	1
1	0	1
1	0	1

D5

1	1	0
1	1	0
1	1	0
1	0	1
1	0	1
1	0	1

Latent variance regimes Z

For $k = 3$, each Z is diagonal and is fully specified by its diagonal variance vector (Z_{11}, Z_{22}, Z_{33}). The complete set of Z variants used is:

- `Z1`: $(1, 1, 1)$
- `Z2_dom_on_1`: $(1, 0.5, 0.5)$
- `Z2_dom_on_2`: $(0.5, 1, 0.5)$
- `Z2_dom_on_3`: $(0.5, 0.5, 1)$
- `Z3_top_mid_bottom`: $(1, 0.5, 0.25)$
- `Z3_top_bottom_mid`: $(1, 0.25, 0.5)$
- `Z3_mid_top_bottom`: $(0.5, 1, 0.25)$
- `Z3_mid_bottom_top`: $(0.5, 0.25, 1)$
- `Z3_bottom_top_mid`: $(0.25, 1, 0.5)$
- `Z3_bottom_mid_top`: $(0.25, 0.5, 1)$

Scenario listing (combinations of D, Z, and j)

Table 1: Scenario library for k=3. Each row specifies a choice of D, a choice of Z, and the target process index j.

D	Z	j
D1	Z1	1
D1	Z1	2
D1	Z1	3
D1	Z2_dom_on_1	1
D1	Z2_dom_on_1	2
D1	Z2_dom_on_1	3
D1	Z2_dom_on_2	1
D1	Z2_dom_on_2	2
D1	Z2_dom_on_3	3
D1	Z3_bottom_top_mid	1

D1	Z3_mid_bottom_top	2
D1	Z3_mid_bottom_top	3
D1	Z3_mid_top_bottom	1
D1	Z3_mid_top_bottom	2
D1	Z3_top_bottom_mid	3
D1	Z3_top_mid_bottom	1
D1	Z3_top_mid_bottom	2
D1	Z3_top_mid_bottom	3
D2	Z1	1
D2	Z2_dom_on_1	1
D2	Z2_dom_on_2	2
D2	Z3_bottom_top_mid	1
D2	Z3_mid_top_bottom	1
D2	Z3_top_mid_bottom	1
D3	Z1	1
D3	Z2_dom_on_1	1
D3	Z2_dom_on_2	2
D3	Z3_bottom_top_mid	1
D3	Z3_mid_top_bottom	1
D3	Z3_top_mid_bottom	1
D4	Z1	1
D4	Z1	2
D4	Z2_dom_on_1	1
D4	Z2_dom_on_1	2
D4	Z2_dom_on_2	1
D4	Z2_dom_on_2	2
D4	Z3_bottom_top_mid	1
D4	Z3_mid_bottom_top	2
D4	Z3_mid_top_bottom	1
D4	Z3_mid_top_bottom	2
D4	Z3_top_mid_bottom	1
D4	Z3_top_mid_bottom	2
D5	Z1	1
D5	Z1	2
D5	Z2_dom_on_1	1
D5	Z2_dom_on_1	2
D5	Z2_dom_on_2	1
D5	Z2_dom_on_2	2
D5	Z3_bottom_top_mid	1
D5	Z3_mid_bottom_top	2
D5	Z3_mid_top_bottom	1
D5	Z3_mid_top_bottom	2
D5	Z3_top_mid_bottom	1
D5	Z3_top_mid_bottom	2

Scenario library for $k = 4$ (with $p = 6$ traits)

Process–trait maps D

`D1`

1	1	1	1
1	1	1	1
1	1	1	1
1	1	1	0
1	1	0	0
1	0	0	0

D2

1	0	0	0
1	0	0	0
0	1	0	0
0	1	0	0
0	0	1	0
0	0	0	1

D3

1	0	1	0
1	0	0	1
1	1	0	0
0	1	0	1
0	1	1	0
0	0	1	1

D4

1	1	0	1
1	1	0	1
1	1	1	0
1	1	1	0
1	0	1	1
1	0	1	1

D5

1	1	0	0
1	1	0	0
1	0	1	0
1	0	1	0
1	0	0	1
1	0	0	1

Latent variance regimes Z

For $k = 4$, each Z is diagonal and is fully specified by its diagonal variance vector $(Z_{11}, Z_{22}, Z_{33}, Z_{44})$. The complete set of Z variants used is:

- `Z1`: $(1, 1, 1, 1)$
- `Z2_dom_on_1`: $(1, 0.5, 0.5, 0.5)$
- `Z2_dom_on_2`: $(0.5, 1, 0.5, 0.5)$
- `Z2_dom_on_3`: $(0.5, 0.5, 1, 0.5)$

- `Z2_dom_on_4`: $(0.5, 0.5, 0.5, 1)$
- `Z3_top_mid_bottom`: $(1, 0.5, 0.5, 0.25)$
- `Z3_top_bottom_mid`: $(1, 0.25, 0.5, 0.5)$
- `Z3_mid_top_bottom`: $(0.5, 1, 0.5, 0.25)$
- `Z3_mid_bottom_top`: $(0.5, 0.25, 0.5, 1)$
- `Z3_bottom_top_mid`: $(0.25, 1, 0.5, 0.5)$
- `Z3_bottom_mid_top`: $(0.25, 0.5, 0.5, 1)$

Scenario listing (combinations of D, Z, and j)

Table 2: Scenario library for k=4. Each row specifies a choice of D, a choice of Z, and the target process index j.

D	Z	j
D1	Z1	1
D1	Z1	2
D1	Z1	3
D1	Z1	4
D1	Z2_dom_on_1	1
D1	Z2_dom_on_1	2
D1	Z2_dom_on_1	3
D1	Z2_dom_on_1	4
D1	Z2_dom_on_2	1
D1	Z2_dom_on_2	2
D1	Z2_dom_on_3	3
D1	Z2_dom_on_4	4
D1	Z3_bottom_top_mid	1
D1	Z3_mid_bottom_top	2
D1	Z3_mid_bottom_top	3
D1	Z3_mid_bottom_top	4
D1	Z3_mid_top_bottom	1
D1	Z3_mid_top_bottom	2
D1	Z3_top_bottom_mid	3
D1	Z3_top_bottom_mid	4
D1	Z3_top_mid_bottom	1
D1	Z3_top_mid_bottom	2
D1	Z3_top_mid_bottom	3
D1	Z3_top_mid_bottom	4
D2	Z1	1
D2	Z2_dom_on_1	1
D2	Z2_dom_on_2	2
D2	Z3_bottom_top_mid	1
D2	Z3_mid_top_bottom	1
D2	Z3_top_mid_bottom	1
D3	Z1	1
D3	Z2_dom_on_1	1
D3	Z2_dom_on_2	2

D3	Z3_bottom_top_mid	1
D3	Z3_mid_top_bottom	1
D3	Z3_top_mid_bottom	1
D4	Z1	1
D4	Z1	2
D4	Z2_dom_on_1	1
D4	Z2_dom_on_1	2
D4	Z2_dom_on_2	1
D4	Z2_dom_on_2	2
D4	Z3_bottom_top_mid	1
D4	Z3_mid_bottom_top	2
D4	Z3_mid_top_bottom	1
D4	Z3_mid_top_bottom	2
D4	Z3_top_mid_bottom	1
D4	Z3_top_mid_bottom	2
D5	Z1	1
D5	Z1	2
D5	Z2_dom_on_1	1
D5	Z2_dom_on_1	2
D5	Z2_dom_on_2	1
D5	Z2_dom_on_2	2
D5	Z3_bottom_top_mid	1
D5	Z3_mid_bottom_top	2
D5	Z3_mid_top_bottom	1
D5	Z3_mid_top_bottom	2
D5	Z3_top_mid_bottom	1
D5	Z3_top_mid_bottom	2
